# Prevalence of HCV among people who inject drugs in Brussels—a respondent-driven sampling survey

**DOI:** 10.1186/s12954-020-00358-3

**Published:** 2020-02-21

**Authors:** Luk Van Baelen, Els Plettinckx, Jérôme Antoine, Lies Gremeaux

**Affiliations:** Sciensano, Rue Juliette Wytsmanstraat, 14, 1050 Brussels, Belgium

**Keywords:** People who inject drugs, Hepatitis C, Belgium, Prevalence

## Abstract

**Background:**

In Belgium, people who inject drugs (PWID) are at a high risk of being infected by hepatitis C (HCV) as injecting drug use is the main mode for transmission of HCV in Europe. Estimates about the number of people living with HCV in Belgium are rare and even less is known about the prevalence of HCV among PWID.

**Method:**

Between 1 February 2019 and 26 April 2019, PWID and high-risk opiate users (HROU) were recruited in Brussels through respondent-driven sampling (RDS). They were invited to a questionnaire and underwent a rapid HCV test.

**Results:**

A total of 253 respondents participated in the study, of which 168 were PWID and 238 were HROU, with 153 respondents belonging to both categories. The overall unweighted sample average for HCV antibodies was 41.1%. The weighted population estimates were 43.7% (95% CI 30.6–56.8%) for RDS-II and 43.4% (95% CI 28.9–58.0%) for RDS-SS.

**Conclusions:**

This prevalence is lower than the prevalence estimates reported elsewhere in Europe. However, the data still suggest that serious efforts are needed to reach the goal set by the WHO to reduce HCV by 2030 with 90%.

## Background

Globally, an estimated 71 million people have a chronic hepatitis C virus (HCV) infection [1]. In the EU and Norway, the total number is estimated at 31,178 HCV cases for 2017. This corresponds to a crude rate of 7.3 cases per 100,000 population [2]. It is estimated that about 54–86% of those infected with HCV will develop chronic HCV [3]. Chronic HCV can lead to cirrhosis, hepatocellular carcinoma, and premature death when it is not treated [1, 4, 5]. Infected persons do not show explicit symptoms of liver disease, but they are often tired and have poor appetite, nausea, muscle ache, etc. [4]. Consequently, a lot of people do not know that they are infected by the virus without undergoing a HCV screening [6]. Moreover, people can be re-infected with the same or different strains of the virus, even after a successful treatment [7]. Over the last 15 years, global mortality related to chronic HCV infection has steadily increased to over 400,000 deaths annually [1]. This contrasts with the declining number of deaths estimated from other infectious diseases such as HIV, tuberculosis, and malaria [1], making HCV one of the main causes of chronic liver disease and mortality worldwide [8].

People who inject drugs (PWID) are at a high risk of being infected by the virus [8, 9], and injecting drug use is nowadays the main mode of HCV transmission [5]. The number of countries with studies quantifying the prevalence of HCV infection among PWID is increasing [10]. Globally, it is estimated that 10 million PWID are infected by HCV [8]. In addition, 23% of new HCV infections and one in three HCV deaths are attributable to injecting drug use [10]. In the EU and Norway, injecting drug use is reported as the likely cause for 40% of acute cases and 55% of chronic cases among the cases for which information on the transmission mode is available [2]. During 2016–2017, HCV antibody prevalence ranged in Europe from 15 to 82% among PWID [11].

Estimates about the number of people living with HCV in Belgium are rare, and even less is known about the prevalence of HCV among PWID [12]. We conducted a sero-behavioral study in Brussels among current PWID to obtain recent and accurate prevalence data of HCV.

## Methods

Our formative research taught us that particularly for Brussels, very few studies were done in the past among the PWID population. Based on expert opinions from key stakeholders including PWID and service providers, the PWID population in Brussels was estimated at between 500 and 1000, but apart from treatment centers and other community-based supportive groups, there were no users groups of PWID, and the connections between PWID were supposed to be weak. As standard probability methods are generally difficult to apply to hard-to-reach populations such as PWID [13] and because the hidden nature of injecting drug use decreased the potential for success of other survey strategies such as time-location sampling or simple random sampling, respondent-driven sampling (RDS) was chosen as the sampling method. It was decided to include also high-risk opiate users (HROU) in the study. They allowed us to reach PWID with a more diverse profile. The HROU, defined following the directives of the EMCDDA as people who used opiates at least once a week for 6 months in the last year without medical prescription [14], were seen as a bridge between different PWID sub-populations or individual PWID.

Between 1 February 2019 and 26 April 2019, we recruited PWID and HROU in Brussels using the RDS methodology. Eligibility criteria were (i) self-reported injecting drug use in the last year and/or opiate use at least once a week for 6 months in the last year without medical prescription, (ii) aged 18 or older, (iii) having lived or used drugs in Brussels, (iv) having received a coupon from someone who participated already or being selected by one of the participating organizations, (v) willingness to answer to a questionnaire, (vi) willingness to undergo a rapid HCV test, and (vii) not having participated in the study before. Interviews were conducted in Dutch, French, or English or in the case of native Arabic- and Russian-speaking interviewers also in these two languages.

Sampling started with seven seeds, selected by low-threshold treatment centers or needle exchange programs in Brussels. Although seeds were selected for convenience, we encouraged diversity based on gender, age, and mode of drug use as usual when applying RDS [15–17]. The recruitment followed the usual RDS sampling method of chain referral, where seeds recruited “first wave” participants and “first wave” participants recruited “second wave” participants, and so on, until the end of the study period. Interviewers checked for track marks to verify recent injecting behavior. If marks were not found or the user confirmed no injecting practices, the subject had to demonstrate detailed acquaintance with opioid preparation for other modes of administration. At the end of the interview, every respondent received three recruitment coupons. The recruitment coupons were valid for 1 week, although expired coupons were not rejected in practice. The limitation of 1 week was mainly applied to encourage participants not to wait too long before introducing the coupons to potential new participants. 90.4% of all participants arrived within this time limit. Because an adequate strategy on non-response or refusals was lacking, no further information is available on the actual response rate, i.e., the number of people who refused to participate. To ensure tracking of subsequent waves, the coupons had a unique identifying number to link the recruiter to the recruited person [18]. Each individual received €5 for participating in the interview and the HCV test and €10 for each eligible participant they recruited, with a maximum of three new recruits per participant. To avoid doubles, we encoded individuals with their initials, sex, and date of birth and used the custom-developed coupon manager software. If seeds turned out not to be productive or the recruitment stopped, we recruited additional seeds. With this strategy, we responded to poor recruitment during data collection without negatively impacting the theoretical and methodological requirements [19]. A team of multi-ethnic and multilingual nurses conducted structured interviews and HCV-testing in a mobile unit. The interviews were immediately imputed in a questionnaire with all mandatory questions, developed in LimeSurvey [20] and saved on a central server. As a result, the sample did not contain missing values.

During the study period, participants could come every day (7/7) between 17.00 and 21.00 to this mobile unit. Initially, it was decided to park the mobile unit on fixed places, every day of the week another place where PWID could come and participate in the study and to repeat these patterns week after week. However, it turned out that the PWID were less mobile than expected, and after ten days, we changed the strategy and parked the mobile unit for several days in a row on a location known to PWID where we stayed until no new recruits arrived anymore. In total, people were interviewed and tested on nine different locations in the city.

At the onset of the interview, participants were informed about the study aims and were asked to sign a consent form. We asked questions on socio-demographic characteristics, drug use and injecting history, HCV risk behavior, previous HCV testing, previous and current HCV treatment, previous and current drug use treatment, number of overdoses, network size, and the relationship with their recruiter. To distinguish between respondents who were rather in contact with PWID and others who were more in contact with HROU, network size was defined as (i) “How many people in Brussels who have injected opiates such as heroin, morphine, opium, or fentanyl in the last 6 months do you know by name who know your name?”; (ii) “How many people in Brussels who have used opiates such as heroin, morphine, opium or fentanyl without prescription and without injecting and who have injected drugs in the last 6 months but not opiates do you know by their name who know your name?”; (iii) “How many people in Brussels who have injected drugs in the last 6 months but not opiates do you know by their name who know your name?”; or (iv) “How many people in Brussels who have used opiates such as heroin, morphine, opium or fentanyl without medical prescription without injecting in the last 6 months do you know by their name who know your name?”. The questionnaire was pre-tested and based on the drug-related infectious disease toolkit, developed by the European Monitoring Centre for Drugs and Drug Addiction (EMCDDA) [21], the WHO guidelines for RDS [22], and the Belgian HIV register (for the unique person’s identifier) [23], with some additional questions from the Australian national drug strategy household survey [24, 25] and a HIV/HCV risk behavior survey from Yale University [26]. Prevalence of HCV was tested with the whole blood InTec Rapid HCV antibody test. We did not have the possibility to confirm HCV antibody positive results with a PCR HCV RNA test as recommended by the national guidelines [6]. Respondents were offered pre- and post-counseling according to the national and international guidelines [6].

Based on the aforementioned estimated number of PWID in Brussels and with an estimated HCV prevalence of 40% [27], a CI of 95%, *z* = 1.96, a design effect of 1.5 [1], and a standard error of 0.05, we reached a sample size on which we applied a finite population correction [28], resulting in a required sample size of 269 PWID. Seeds were included in every part of the analysis. Selection bias was examined for age and sex by comparing the participants’ profile with the profile of patients in low-threshold treatment centers and needle exchange programs in Brussels and within the sample through *t* tests and *χ*^2^ tests.

RDSAT version 7.1.46 was used to verify the stability of the study sample. The homophily metric (Hx) was between − 1 and 1, with Hx < − 0.3 and Hx > 0.3 used for the identification of significant biases, as is conventional in RDS analysis [18, 29]. We used RStudio version 1.1.442, package “RDS” [30] to calculate equilibrium for sex, mean age, and HCV prevalence, which was attained when the sample distribution from one recruitment wave to the next fell within a discrepancy of less than 2% [28]. For respondents who answered that their personal network size was 0, degree values were imputed using the weighted mean of the non-missing degrees, calculated with Gile’s SS [30]. Since there is no consensus on which estimator is optimal [31], we give the unweighted sample average for HCV antibody prevalence among PWID as well as the RDS-II and RDS-SS weighted estimate. We did the graphical representation of the complete network in NetDraw version 2.160. For reasons of completeness, descriptive statistics for HROU will be presented as well. The reporting of this study conforms to the STROBE guidelines for RDS studies [32].

## Results

**Fig. 1:**
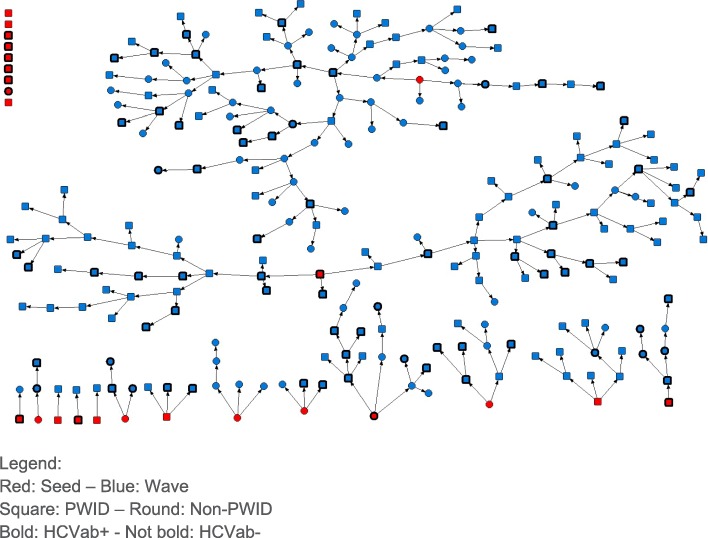
Recruitment chains of RDS in Brussels, 2019. Red, seed; blue, PWID, round; non-PWID, bold; HCVab+, not bold, HCVab−

Ultimately, a total of 256 people were considered eligible, consented, and were recruited. The RDS chain referral started with 7 seeds, with an additional 14 seeds that were selected when recruitment stopped. Seeds were mainly men (95.2%), living in Brussels (95.2%), and having a median age of 42 and a negative HCV antibody status (57.1%). In addition, two eligible recruits were selected by a respondent who was not eligible, and one participant managed to refer four new respondents instead of the allowed three. We decided to keep the data for these three additional participants as separate seeds, which gives an overall number of 24 seeds. These 24 seeds recruited 229 eligible respondents, which result in a total of 253 respondents and a response proportion of 30%. Fifteen seeds resulted in at least one wave of new participants. Two seeds resulted in the two longest chains that were together responsible for the participation of 172 respondents or 68% of all respondents. In total, we have an average of 3.3 waves per seed (± SD 3.2, median 2, range 1–11). Three respondents managed to participate twice, which was discovered only after the interview took place. The data for their second participation were excluded from the analysis, and they did not recruit any new respondents. Of the 253 respondents, 168 were PWID, and 238 were HROU, with 153 respondents belonging to both categories. The recruitment chains are presented in Fig. 1. After 3 months, the targeted community of PWID and HROU in Brussels was saturated.

For HCV antibody prevalence and age, the number of waves required for equilibrium was five, whereas for recent injecting behavior and sex, it was nine. Homophily among participants was overall positive, except for female respondents (Hx = − 0.27) and respondents who were HCV antibody positive (Hx = − 0.01). Recruitment weights for the full sample ranged between 0.93 and 1.08 for age, 0.98 and 1.23 for sex, 0.99 and 1.15 for recent non-prescribed opioid use, 0.98 and 1.01 for recent injecting behavior, and 0.96 and 1.02 for HCV antibody status.

The overall unweighted sample average for HCV antibodies was 41.1%. The weighted population estimates were 43.7% (95%CI 30.6–56.8%) for RDS-II and 43.4% (95%CI 28.9–58.0%) for RDS-SS. As shown in Table 1, PWID were mostly male (90.5%) and living in Brussels (92.9%), with a median age of 40 years. When comparing the PWIDs’ profile with the profile of patients in low-threshold treatment centers and needle exchange programs in Brussels, age did not significantly differ (*t* = − 0.45, *p* = 0.65). However, we had a lower proportion of women in the sample compared with patients known to the established structures (*χ*^2^ = 6.2, *p* = 0.013). Indeed, the sample only contained 9.5% female PWID, whereas the other structures had between 17.8% and 18.5% women. Among PWID in the sample, there were no significant differences between men and women for age (*t* = − 0.31, *p* = 0.76), injection of heroin (*χ*^2^ = 0.13, *p* = 0.94), injection of cocaine (*χ*^2^ = 1.7, *p* = 0.2), recruiter (*χ*^2^ = 6.2, *p* = 0.18), or other variables of interest. However, the unweighted sample average for HCV antibody for female PWID was 81.3% (95%CI 57.0–93.4%), whereas the unweighted HCV antibody sample estimate for male PWID was 36.8% (95%CI 29.6–44.7%; *χ*^2^ = 10.03, *p* = 0.002).

**Table 1:** Descriptive statistics for PWID and HROU in Brussels, 2019

			PWID and HROU (a)		PWID non-HROU (b)		Non-PWID HROU(c)		Total PWID (a)+(b)		Total HROU (a)+(c)	
Age (mean ± sd, median, range)			40.5 ± 9.5, 40, 20–65		37.0 ± 8.6, 34, 23–52		41.7 ± 9.2, 45, 19–65		40.1 ± 9.4, 40, 20–65		40.9 ± 9.4, 42, 19–65	
			*N*	%	*N*	%	*N*	%	*N*	%	*N*	%
Sex	Male		139	90.9	13	86.7	80	94.1	152	90.5	219	92.0
	Female		14	9.2	2	13.3	5	5.9	16	9.5	19	8.0
Place of residence	In Brussels		145	94.8	11	73.3	82	96.5	156	92.9	227	95.4
	Not in Brussels		8	5.2	4	26.7	3	3.5	12	7.1	11	4.6
Use of not-prescribed opiates at least once a week for 6 months in the last 12 months	Yes		153	100.0	0	0.0	85	100.0	153	91.1	238	100.0
	No		0	0.0	15	100.0	0	0.0	15	8.9	0	0.0
Injected any drug in the last 12 months	Yes		153	100.0	15	100.0	0	0.0	168	100.0	153	64.3
	No		0	0.0	0	0.0	85	100.0	0	0.0	85	35.7
Injected heroin in the last 12 months	Yes		120	78.4	7	46.7	0	0.0	127	75.6	120	50.4
	No		33	21.6	8	53.3	85	100.0	41	24.4	118	49.6
Injected cocaine in the last 12 months	Yes		125	81.7	13	86.7	0	0.0	138	82.1	125	52.5
	No		28	18.3	2	13.3	85	100.0	30	17.9	113	47.5
Injected other opiates in the last 12 months	Yes		54	35.3	2	13.3	0	0.0	56	33.3	54	22.7
	No		99	64.7	13	86.7	85	100.0	112	66.7	184	77.3
Injected amphetamines in the last 12 months	Yes		21	13.7	6	40.0	0	0.0	27	16.1	21	8.8
	No		132	86.3	9	60.0	85	100.0	141	83.9	217	91.2
Injected NPS in the last 12 months	Yes		9	5.9	0	0.0	0	0.0	9	5.4	9	3.8
	No		144	94.1	15	100.0	85	100.0	159	94.6	229	96.2
Injected other drugs in the last 12 months	Yes		6	3.9	1	6.7	0	0.0	7	4.2	6	2.5
	No		147	96.1	14	93.3	85	100.0	161	95.8	232	97.5
Ever tested on HCV	Yes		98	64.1	7	46.7	47	55.3	105	62.5	145	60.9
		Infected	*51*	*52.0*	*4*	*57.1*	*8*	*17.0*	*55*	*52.4*	*59*	*40.7*
		Not infected	*43*	*43.9*	*2*	*28.6*	*37*	*78.7*	*45*	*42.9*	*80*	*55.2*
		Do not know	*4*	*4.1*	*1*	*14.3*	*2*	*4.3*	*5*	*4.8*	*6*	*4.1*
	No		51	33.3	8	53.3	28	32.9	59	35.1	79	33.2
	Don't know		4	4.1	0	0.0	10	11.8	4	2.4	14	5.9
Ever in treatment for HCV (only for those who were tested and infected)	Yes		20	39.2	1	25.0	3	37.5	21	38.2	23	39.0
	No		31	60.8	3	75.0	5	62.5	34	61.8	36	61.0
Ever in substitution treatment	Yes		120	78.4	9	60.0	60	70.6	129	76.8	180	75.6
		Now	*87*	*72.5*	*5*	*55.6*	*43*	*71.7*	*92*	*71.3*	*130*	*72.2*
		Before	*33*	*27.5*	*4*	*44.4*	*17*	*28.3*	*37*	*28.7*	*50*	*27.8*
	No		33	21.6	6	40.0	24	28.2	39	23.2	57	24.0
	Do not know		0	0.0	0	0.0	1	1.2	0	0.0	1	0.4
Ever in other drug-related treatments	Yes		82	53.6	8	53.3	36	42.4	90	53.6	118	49.6
		Now	*17*	*20.7*	*0*	*0.0*	*5*	*13.9*	*17*	*19.1*	*22*	*18.8*
		Before	*64*	*78.0*	*8*	*100.0*	*31*	*86.1*	*72*	*80.9*	*95*	*81.2*
	No		71	46.4	7	46.7	49	57.6	78	46.4	120	50.4
Referral	Stranger		14	9.2	3	20.0	11	12.9	17	10.1	25	10.5
	Acquaintance but not close		54	35.3	5	33.3	33	38.8	59	35.1	87	36.6
	Friend		63	41.2	6	40.0	18	21.2	69	41.1	81	34.0
	Partner		6	3.9	0	0.0	2	2.4	6	3.6	8	3.4
	Family		0	0.0	0	0.0	3	3.5	0	0.0	3	1.3
	Dealer		1	0.7	0	0.0	0	0.0	1	0.6	1	0.4
	Seeds		15	9.8	1	6.7	8	9.4	16	9.5	23	9.7
Risk behavior (past 30 days)	Split drugs with someone else		85	55.6	7	46.7	0	0.0	92	54.8	85	55.6
	Used a syringe that had been used before		40	26.1	5	33.3	0	0.0	45	26.8	40	26.1
	Injected drugs that were mixed, measured or divided up with a used syringe		72	47.1	6	40.0	0	0.0	78	46.4	72	47.1
	Draw a shot from someone else’s cooker		72	22.9	7	46.7	0	0.0	79	47.0	72	47.1
	Draw a shot from someone else’s water		60	39.2	6	40.0	0	0.0	66	39.3	60	39.2
	Used rinse water from somebody else		39	25.5	4	26.7	0	0.0	43	25.6	39	25.5
HCV antibody test	Positive		66	43.1	3	20.0	14	16.5	69	41.1	80	33.6
	Negative		87	56.9	12	80.0	71	83.5	99	58.9	158	66.4
Total			**153**	**100**	**15**	**100**	**85**	**100**	**168**	**100**	**238**	**100**

Most PWID injected heroin (75.6%), cocaine (82.1%), or other opiates (33.3%) in the last year. In addition, 91.1% of the PWID used not-prescribed opiates at least once a week for 6 months in the last 12 months. Almost three in four reported to have ever received opioid substitution treatment (76.8%), while among them, 71.3% were currently in opioid substitution treatment. Most PWID had been tested in the past for HCV (62.5%), and among them, 52.4% reported a positive test result. Only 21 respondents, or 38.2% from those who said they were infected with HCV, reported they had been treated for HCV in the past.

The relationship between recruiters and recruit was as follows: 41.1% of the respondents were recruited by a friend, 35.1% by an acquaintance with whom they were not close, and 10.1% by a stranger. The reported network size ranged from 0 to 1000 individuals, with an average of 37 (± SD 120.6, median 10). Other additional information which is not shown in Table 1 is as follows: out of 159 PWID who had used non-prescribed opiates in the last year, 32 (or 20%) reported an overdose due to opiates. Out of 146 recent PWID who reported previous or current treatment for their drug use, only 97 (or 66.4%) said they had been tested for HCV before, whereas out of 20 ever PWID who reported previous or current treatment for their drug use, 17 (or 85%) said they had been tested for HCV before. Recent HCV risk behavior (last 30 days) related to sharing injection equipment among PWID ranged between 25.6 (using rinse water that somebody else had used before) and 54.8% (having split drugs with other persons).

As shown in Table 2, for PWID who tested positive for HCV antibodies, the odds were higher when they were female, they had already injected other opiates than heroin, they had been tested before for HCV, and they were on substitution treatment. Interestingly, drawing a shot from a cooker in the past 30 days that was used by someone else before decreased the odds for a positive HCV antibody result, although this result was only slightly statistically significant and might be an artifact. In that case, there would be no association between recent risk behavior and a positive HCV antibody test result.

**Table 2:** Regression estimates, standard error (SE), *z* value, and odds ratios for PWID having tested positive on the HCVab test in Brussels, 2019

	Estimate	SE	*z* value	Odds ratio (95% CI)
(Intercept)	1.52	1.08	1.42	4.59 (0.56–37.89)
Not used heroin in the past 12 months (vs used heroin)	− 1.53	1.03	− 1.49	0.22 (0.03–1.63)
Female (vs male)	2.10	0.84	2.49	**8.17 (1.57–42.6)**
Never injected heroin (vs injected)	− 0.72	0.84	− 0.85	0.49 (0.09–2.54)
Never injected other opiates (vs injected)	− 1.74	0.51	− 3.38	**0.18 (0.06–0.48)**
Never injected amphetamines (vs injected)	− 0.86	0.56	− 1.54	0.42 (0.14–1.26)
Never injected cocaine (vs cocaine)	0.74	1.07	0.69	2.1 (0.26–17.01)
In the past 30 days				
Split drugs with someone else (vs not split drugs)	− 0.39	0.57	− 0.68	0.68 (0.22–2.08)
Used a syringe that had been used before (vs not used syringe)	1.45	0.88	1.65	4.25 (0.76–23.84)
Injected drugs that were mixed, measured or divided up with a used syringe (vs not mixed, measured or divided up with a used syringe)	0.55	0.85	0.65	1.73 (0.33–9.09)
Draw a shot from someone else’s cooker (vs not draw shot from other cooker)	− 1.79	0.82	− 2.18	**0**.**17 (0**.**03–0**.**83)**
Draw a shot from someone else’s water (vs not draw shot from other's water)	− 0.10	0.80	− 0.13	0.9 (0.19–4.3)
Used rinse water from somebody else (vs not used other’s rinse water)	0.52	0.71	0.73	1.68 (0.42–6.76)
Not tested for HCV before (vs tested for HCV before)	− 1.98	0.56	− 3.52	**0**.**14 (0**.**05–0**.**42)**
Not on substitution treatment (vs on substitution treatment)	− 2.33	0.70	− 3.34	**0**.**1 (0**.**02–0**.**38)**
Not on other drug treatment (vs on other drug treatment)	0.84	0.48	1.75	2.33 (0.91–5.97)

## Discussion

In the Brussels context, the recruitment strategy through RDS turned out to be useful to collect data among PWID and HROU. Although almost all of the PWID reported regular use of not-prescribed opiates in the last year, this study reached a substantial proportion of PWID (45.2%) who were currently not on opiate substitution treatment. Also, 35.1% of the PWID indicated to have never been tested for HCV before.

The study gives important results about HCV among PWID. Firstly, approximately 43% of the PWID in Brussels tested positive for HCV antibodies. This prevalence is lower compared with prevalence estimates reported in Europe. According to a European systematic review in 2014 [5], the median number of positive HCV antibodies among PWID tested for HCV in Europe was 82.9% (IQR 59–100%), and figures for 2019 reported that in eight out of the 14 countries in Europe with the national data, more than half of the PWID have been infected with HCV [11]. The lower prevalence in Brussels might be the result of considerable efforts to reduce harms among PWID. In Brussels, there are five needle exchange programs on seven locations, and additionally, pharmacies are involved in the distribution of kits at the cost of 0.5 euro, containing two syringes, two alcohol swabs, two dry post-injecting swabs, two spoons, two flasks of injectable sterile water, and harm reduction information. These programs already exist for more than 20 years in Brussels.

Secondly, previous research in Belgium found that 86.5% of recent PWID and 84.7% of every PWID in treatment between 2011 and 2014 were tested for HCV between 2008 and 2015 [12]. In the current study, only 66.4% of recent PWID who reported previous or current treatment for their drug use said they had been tested for HCV before. This is much lower than the results from the previous study indicated [12]. However, 85% of every PWID who reported previous or current treatment for their drug use said they had been tested for HCV before, which corresponds to what was found in the previous study [12]. These results could indicate that the recent PWID in the current study were much earlier in their drug career than the recent PWID in the previous study and that PWID have to spend some time in treatment before they are tested for HCV. As described in previous research, the lack of knowledge and incorrect assumptions among health professionals working with this population can hamper successful HCV diagnoses and treatment [33, 34].

It is also important to mention the limitations of this study. Firstly, although the results clearly indicate that HCV is endemic among the study population, this study does not provide evidence about whether HCV is attributed to drug use rather than sex-related or other behavior [9]. Indeed, the data are not appropriate for determining what led to the HCV infection. For example, recent risk behavior (last 30 days) did not increase the odds for a positive HCV antibody result. Only characteristics that indicate a longer injection career such as being on substitution treatment or having been tested before on HCV, as well as being female and having injected other opiates than heroin in the last 12 months increased the odds for a positive test result. However, based on this information, it remains unclear what caused the HCV infection.

Secondly, because of budgetary and logistical constraints, we could not reach the required sample size of 269. Given the small total PWID population in Brussels, we have tried to overcome this weakness by comparing the characteristics for the sample with the profile of PWID in other databases from treatment centers and a crisis intervention center in Brussels. However, we cannot exclude that some subpopulations were not included in the final sample. For example, notwithstanding tremendous efforts, we could not reach a recruitment chain of people who intentionally use and inject drugs to have sex—chemsex—among men who have sex with men. In addition, some Arab- and Russian-speaking PWID dropped out because they wanted to participate at a moment when there was no interviewer available who spoke their language. Finally, the RDS sampling method resulted in a lower proportion of women compared with female PWID known to the established structures in Brussels. The female PWID in the current study tested significantly more positive on HCV antibodies than the male respondents. A systematic review and meta-analysis of 28 studies published globally found an overall higher risk of HCV infection of 36% for female PWID compared with male PWID [35]. Since the profile of these women in the current study did not vary significantly from men for other variables, it is necessary to study the behavioral or structural factors contributing to sex-related differences more in detail in future research. We could mention two potential explanations for the lower proportion of women. Firstly, sampling bias might be the reason, although it remains unclear why. Secondly, it might be that women find their way to treatment centers more easily than men, and they are overrepresented in established drug-related structures compared to their presence in the street. Previous research shows for example that women search more actively for health-related information [36] and visit more often health-care providers for physical but also mental health concerns compared with men [37, 38]. For men, stigma has a more deterrent effect on help-seeking compared with women [37, 39].

In conclusion, firstly, the current study shows results for the prevalence of HCV which are below the European average, but the data still suggest that serious efforts are needed to reach the goal set by the WHO to reduce HCV by 2030 with 90% [40]. Secondly, RDS turned out to be very useful to reach PWID at the beginning of their drug career who were not yet in contact with treatment centers and who had not yet been tested.

## Data Availability

The datasets used and/or analyzed during the current study are available from the corresponding author on reasonable request.

## Abbreviations

CI: Confidence interval
EMCDDA: European Monitoring Centre for Drugs and Drugs Addiction
HCV: Hepatitis C
HCVab: Hepatitis C antibodies
HROU: High-risk opiate users
Hx: Homophily metric
PWID: People who inject drugs
RDS: Respondent-driven sampling
